# Rosmarinic acid attenuates cardiac fibrosis following long-term pressure overload via AMPKα/Smad3 signaling

**DOI:** 10.1038/s41419-017-0123-3

**Published:** 2018-01-24

**Authors:** Xin Zhang, Zhen-Guo Ma, Yu-Pei Yuan, Si-Chi Xu, Wen-Ying Wei, Peng Song, Chun-Yan Kong, Wei Deng, Qi-Zhu Tang

**Affiliations:** 1 Department of Cardiology, Renmin Hospital of Wuhan University, Wuhan, 430060 China; 2 Cardiovascular Research Institute of Wuhan University, Wuhan, 430060 China; 3 Hubei Key Laboratory of Cardiology, Wuhan, 430060 China

## Abstract

Agonists of peroxisome proliferator-activated receptor gamma (PPAR-γ) can activate 5′ AMP-activated protein kinase alpha (AMPKα) and exert cardioprotective effects. A previous study has demonstrated that rosmarinic acid (RA) can activate PPAR-γ, but its effect on cardiac remodeling remains largely unknown. Our study aimed to investigate the effect of RA on cardiac remodeling and to clarify the underlying mechanism. Mice were subjected to aortic banding to generate pressure overload induced cardiac remodeling and then were orally administered RA (100 mg/kg/day) for 7 weeks beginning 1 week after surgery. The morphological examination, echocardiography, and molecular markers were used to evaluate the effects of RA. To ascertain whether the beneficial effect of RA on cardiac fibrosis was mediated by AMPKα, AMPKα2 knockout mice were used. Neonatal rat cardiomyocytes and fibroblasts were separated and cultured to validate the protective effect of RA in vitro. RA-treated mice exhibited a similar hypertrophic response as mice without RA treatment, but had an attenuated fibrotic response and improved cardiac function after pressure overload. Activated AMPKα was essential for the anti-fibrotic effect of RA via inhibiting the phosphorylation and nuclear translocation of Smad3 in vivo and in vitro, and AMPKα deficiency abolished RA-mediated protective effects. Small interfering RNA against Ppar-γ (siPpar-γ) and GW9662, a specific antagonist of PPAR-γ, abolished RA-mediated AMPKα phosphorylation and alleviation of fibrotic response in vitro. RA attenuated cardiac fibrosis following long-term pressure overload via AMPKα/Smad3 signaling and PPAR-γ was required for the activation of AMPKα. RA might be a promising therapeutic agent against cardiac fibrosis.

## Introduction

Cardiac remodeling, characterized as cardiomyocyte hypertrophy and interstitial fibrosis, is a leading risk factor for heart failure, arrhythmia, and sudden death^1,2^. As a key feature of cardiac remodeling, cardiac fibrosis is defined as superfluous transdifferentiation from cardiac fibroblasts (CFs) to myofibroblasts and a disrupted homeostasis between synthesis and degradation of the extracellular matrix (ECM)^3^. Once activated, CFs phenoconvert into myofibroblasts and secrete ECM. The ECM provides structural support for cardiomyocytes and non-structural components under physiological conditions but leads to cardiac fibrosis with anomalous deposition in the presence of chronic injurious stimuli^4^. Massive ECM accumulation may impair mechanical and electrical functions of the heart and subsequently result in heart failure and arrhythmogenesis^5^. Besides, excessive perivascular ECM may also deform the vasculature and separate myocytes from adjacent capillaries, which limits the blood supply of myocytes^6^. CFs are responsible for orchestrating cardiac fibrosis; thus, effective strategies that targeting CFs may help to develop efficacious interventions against fibrosis, mitigate cardiac remodeling, and postpone the development of heart failure^7^.

Smad3 is identified as a central intracellular mediator of myofibroblasts transdifferentiation and ECM synthesis in the pathogenesis of cardiac fibrosis^8,9^. A previous study indicated that knocking out Smad3 in mice blocked myofibroblasts activation and protected against angiotensin II-induced cardiac fibrosis^10^. Enhanced phosphorylation and nuclear translocation of Smad3 in fibroblasts was largely responsible for the aggravating fibrosis phenotype following myocardial infarction^8^. These results indicated that inhibition of Smad3 might be beneficial for alleviating cardiac fibrosis and that unearthing a negative regulator of Smad3 could be of paramount clinical importance. 5′ AMP-activated protein kinase (AMPK) has been recognized as a key regulator of energy metabolism in the heart; however, current available studies implied that AMPK extended well beyond its energy-regulating function and played a crucial role in regulating cardiac fibrosis^11,12^. AMPK deficiency exacerbated transverse aortic constriction-induced cardiac fibrosis, whereas the activation of AMPK attenuated cardiac fibrosis^11,13^. Moreover, a previous study indicated that the activation of AMPK negatively regulated Smad3 activation and exerted anti-fibrotic effects^14^. Therefore, pharmacological activation of AMPK may be of great therapeutic interest for treating cardiac fibrosis.

Rosmarinic acid (α-o-caffeoyl-3, 4-dihydroxyphenyl lactic acid; RA), a natural poly-phenolic compound, is widely distributed in species of the *Boraginaceae* and subfamily *Nepetoideae* of the *Labiatae*, which include oregano, sage, mint, sweet basil, and perilla, etc^15,16^. Growing interests are raised on RA for its wide spectrum of biological activities, delineated as powerful anti-oxidative, anti-inflammatory, anti-proliferative, anti-bacterial, and human immunodeficiency virus-1-inhibiting properties, etc^17–19^. RA has been reported to regulate mitogen-activated protein kinase (MAPK) and protein kinase B (PKB/AKT) signaling, which are critically involved in cardiac hypertrophy and fibrosis^16,20,21^. Besides, RA could suppress hypertension via regulating angiotensin-converting enzyme^22^ or the endothelium-dependent vasodilator effect^23^. It is noteworthy that RA could activate peroxisome proliferator-activated receptor gamma (PPAR-γ) in hepatic stellate cells and exerted anti-fibrotic effect^24^. Previous studies indicated that agonists of PPAR-γ could be also effective in activating AMPK^25,26^. Based on these findings, we hypothesized that RA may be a promising candidate for the treatment of cardiac hypertrophy and fibrosis.

In the current study, we provided the first evidence of the protective effects and underlying mechanisms of RA on cardiac hypertrophy and fibrosis. We found that RA could attenuate cardiac fibrosis in mice induced by pressure overload with no alteration in hypertrophic phenotype. Mechanistically, we demonstrated that the protective effects of RA on cardiac fibrosis may be attributed to its activation of AMPKα and inhibition of Smad3 in vivo and in vitro.

## Results

### RA attenuated cardiac dysfunction in mice following long-term pressure overload

**Fig. 1:**
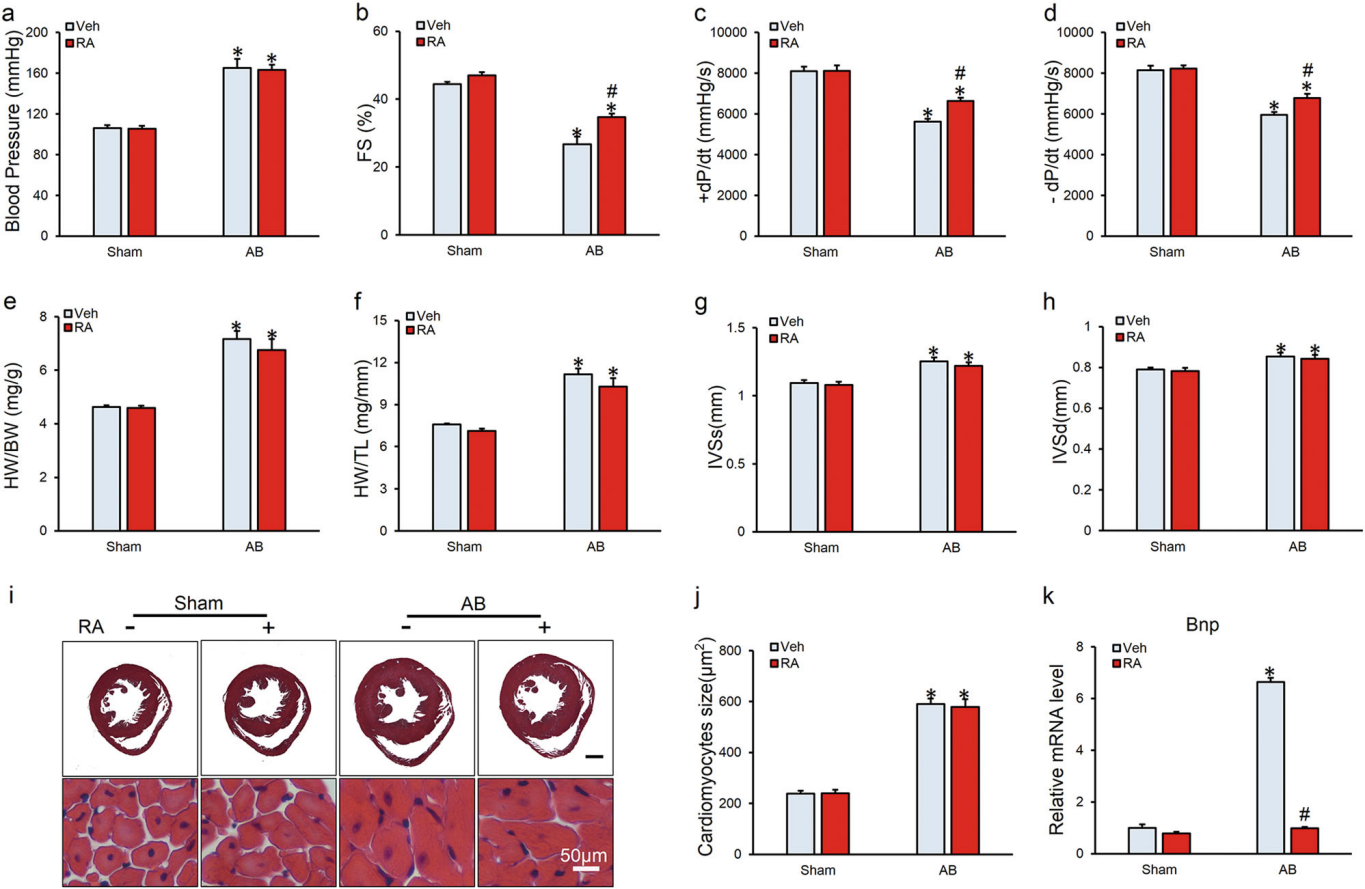
Rosmarinic acid (RA) attenuated cardiac dysfunction in mice following long-term pressure overload **a** The maximum carotid pressure of mice in the four groups (*n* = 13–14). **b** Fractional shortening (FS) of mice as determined via echocardiography at 8 weeks after the AB surgery (*n* = 15). **c,** **d** Hemodynamic analysis of mice with or without RA protection (*n* = 13–14). **e, f** Statistical results of the heart weight (HW)/body weight (BW) and HW/tibia length (TL) (*n* = 15). **g,** **h** Interventricular septal thickness at systole or diastole (IVSs or IVSd) (*n* = 15). **i**, **j** HE staining and statistical results of the cross sectional area (*n* = 6). **k** The relative mRNA level of Bnp normalized to Gapdh in mice (*n* = 6). Values represent the mean ± SEM. *P < 0.05 vs. the corresponding Sham group, ^#^*P* < 0.05 vs. AB+Veh

To clarify whether the beneficial effect of RA on cardiac dysfunction is secondary to its anti-hypertensive effects, we first investigated the blood pressure and found no difference in the maximum carotid artery pressure between vehicle and RA-treated group either at baseline or after the aortic banding (AB) operation (Fig. 1a). Mice exhibited cardiac dysfunction with reduced fractional shortening (FS) and ±dp/dt in response to 8-week pressure overload, which were significantly attenuated after RA treatment (Figs. 1b-d). No alteration in body weight (BW) or heart rate was observed among all groups (Figure S1a-b). We next examined the potential effect of RA on cardiac hypertrophy and observed similar hypertrophic responses in mice with or without RA treatment, as revealed by the similar increases in heart weight (HW)/BW, HW/tibia length (TL), and interventricular septal thickness at systole or diastole (IVSs or IVSd) (Figs. 1e-h). All the echo data are shown in Supplementary Table 1. This observation was further confirmed by the hematoxylin and eosin (HE) staining and cross-sectional area of cardiomyocytes (Figs. 1i, j). In line with these data, the mRNA levels of markers associated with cardiac hypertrophy, including Anp and β-Mhc, showed no significant alterations with or without RA treatment, despite the downregulated mRNA level of brain natriuretic peptide (Bnp) was observed in AB+RA group compared with that of AB group (Figure S1c-d, Fig. 1k). Taken together, these results indicated that RA could attenuate cardiac dysfunction in mice following long-term pressure overload independent of cardiac hypertrophy inhibition.

### RA protected against cardiac fibrosis in vivo

**Fig. 2:**
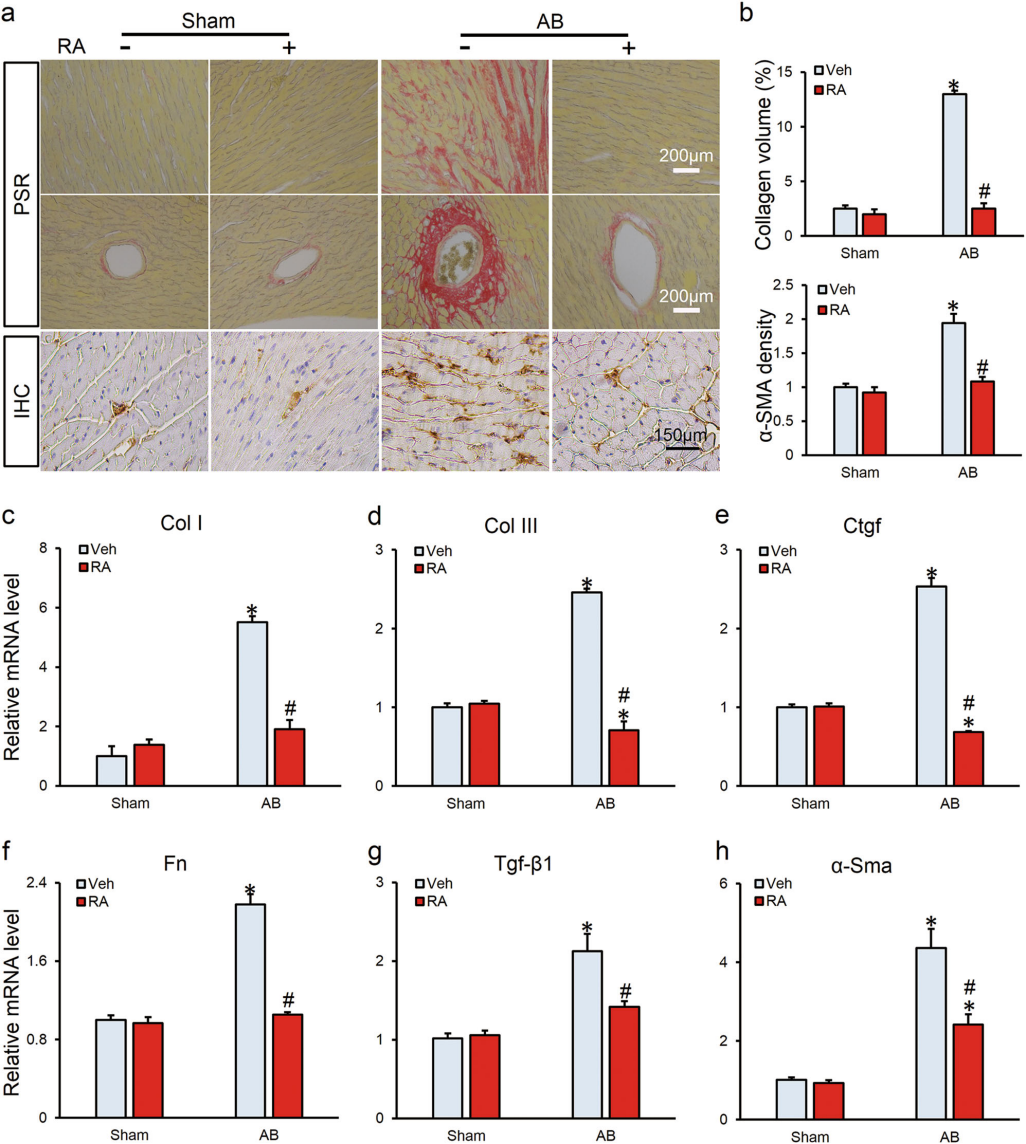
RA protected against cardiac fibrosis in vivo **a** Representative images of PSR staining and immunohistochemical images of α-SMA (*n* = 6). **b** Statistical results of average collagen volume and α-SMA density (*n* = 6). **c**-**h** The relative mRNA levels of fibrotic markers normalized to Gapdh in mice (*n* = 6). Values represent the mean ± SEM. *P < 0.05 vs. the corresponding Sham group, ^#^*P* < 0.05 vs. AB+Veh

As cardiac fibrosis is an essential feature of the cardiac remodeling, and contributes to the conversion from hypertrophy to heart failure by increasing myocardial stiffness and reducing pumping capability^3,5^, we thus detected the effect of RA on cardiac fibrosis. As shown in Figs. 2a, b, RA-treated mice presented with less collagen deposition than that in mice without RA treatment after AB insult, which came in parallel with reduced mRNA levels of fibrotic markers, collagen I (Col I), Col III, connective tissue growth factor (Ctgf), fibronectin (Fn), transforming growth factor-beta (Tgf-β1) and α-smooth muscle actin (α-Sma; Figs. 2c-h). Cardiac fibrosis was further quantified by immunochemistry of α-SMA and we found that RA treatment decreased the expression of α-SMA in hypertrophic hearts (Figs. 2a, b).

Seeing that oxidative stress and inflammation are implicated in the pathogenesis of cardiac fibrosis^27,28^ and RA has been reported to blunt oxidative stress and inflammation via NF-E2-related factor 2 (Nrf2), inducible nitric oxide synthase (iNOS) and cyclooxygenase-2 (COX2) signaling^29–31^, we speculated whether the cardioprotective effect of RA was secondary to alleviated oxidative stress and inflammation. No change in nuclear translocation of Nrf2 or superoxide dismutase 2 (SOD2) expression was observed in mice with or without RA treatment (Figure S2a-c). We previously found that inflammatory response almost returned to normal level at 8 weeks after AB^32^, thus we examined the effect of RA on inflammation 4 weeks after surgery. Unexpectedly, increased expression levels of COX2 and iNOS after AB surgery were unaffected by RA (Figure S2a, d-e), and correspondingly, mRNA level of inflammatory markers exhibited no difference in hypertrophic hearts treated with RA except for Tnf-α (Figure S2f).

### RAactivated AMPKα in vivo and knockout of AMPKα abrogated its protective effects

**Fig. 3:**
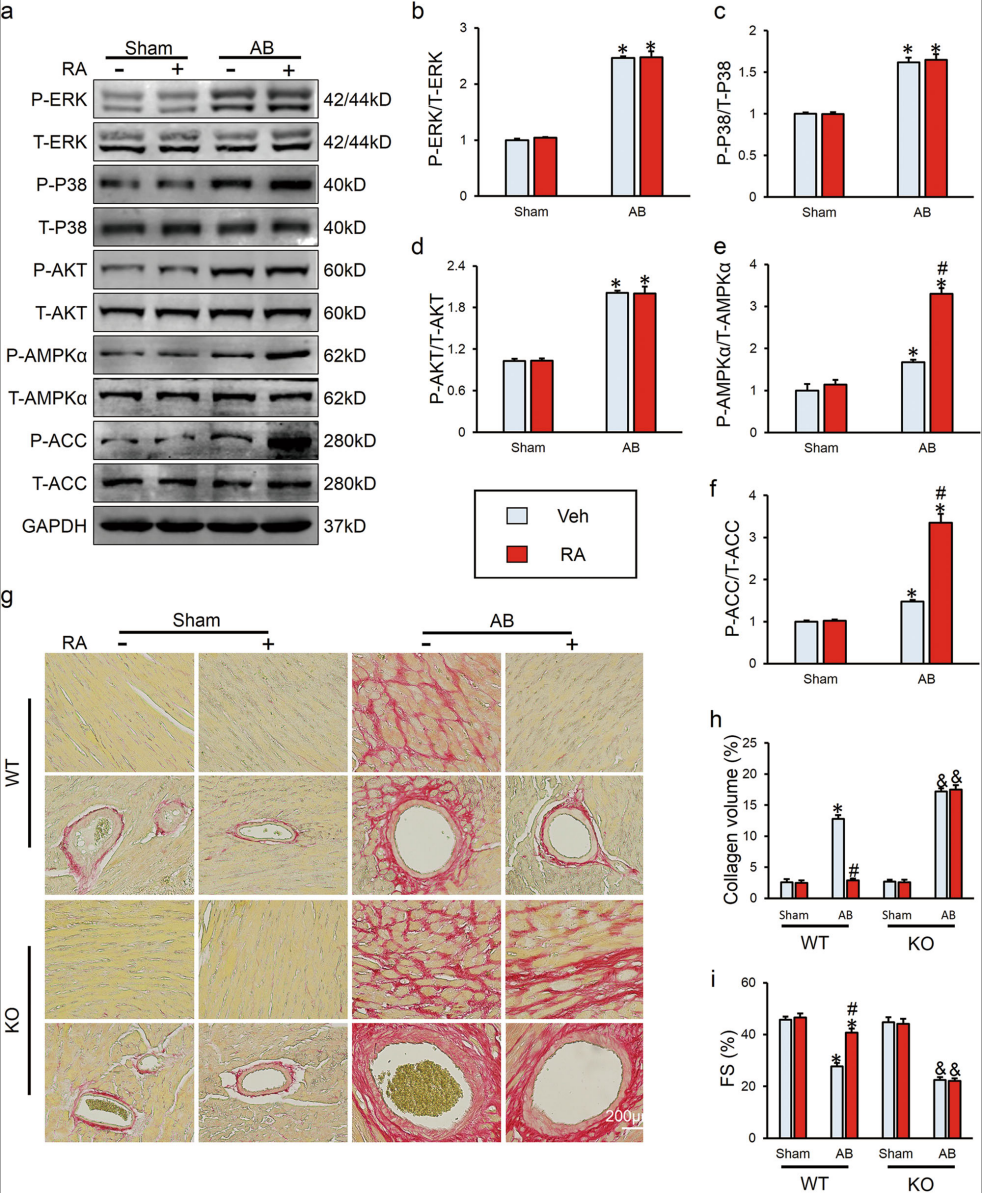
RAactivated AMPKα in vivo and knockout of AMPKα abrogated its protective effects **a**-**f** Representative western blots and quantitative results in wild type (WT) mice (*n* = 6). **g,** **h** PSR staining and statistical results of average collagen volume in WT and AMPKα2 knockout (KO) hearts (*n* = 6). **i** FS in WT and KO mice (*n* = 8–10). Values represent the mean ± SEM. *P < 0.05 vs. the corresponding Sham group within WT mice, ^#^*P* < 0.05 vs. AB+Veh in WT mice, ^&^*P* < 0.05 vs. the corresponding Sham group in KO mice

We further examined the precise mechanism involved in the anti-fibrotic effect of RA. Previous studies indicated that pretreatment with RA could inhibit phosphorylation of MAPKs^16,21,33^ and increase the phosphorylation of AKT^20,34^, therefore we investigated MAPKs and AKT pathway after RA treatment. Unexpectedly, RA did not alter the phosphorylation of extracellular regulated protein kinases (ERK), P38 or AKT (Figs. 3a-d). Agonists of PPAR-γ have been proven to be effective in activating AMPK, and we found that AMPKα was activated by RA in hypertrophic hearts, further validated by increased phosphorylation of ACC, which could reflect a high AMPKα activity (Figs. 3a, e, f). To gain evidence that anti-fibrotic effects of RA were mediated via the activation of AMPKα, we used AMPKα2 knockout (KO) mice. As expected, KO of AMPKα abrogated the protective effects of RA on cardiac fibrosis in response to pressure overload, as evidenced by picrosirius red (PSR) staining (Figs. 3g, h). Correspondingly, ameliorated cardiac function after RA treatment was abolished after the absence of AMPKα (Fig. 3i). All the echo data are shown in Supplementary Table 2.

### RA suppressed Smad3 phosphorylation and nuclear translocation through AMPKα activation

**Fig. 4:**
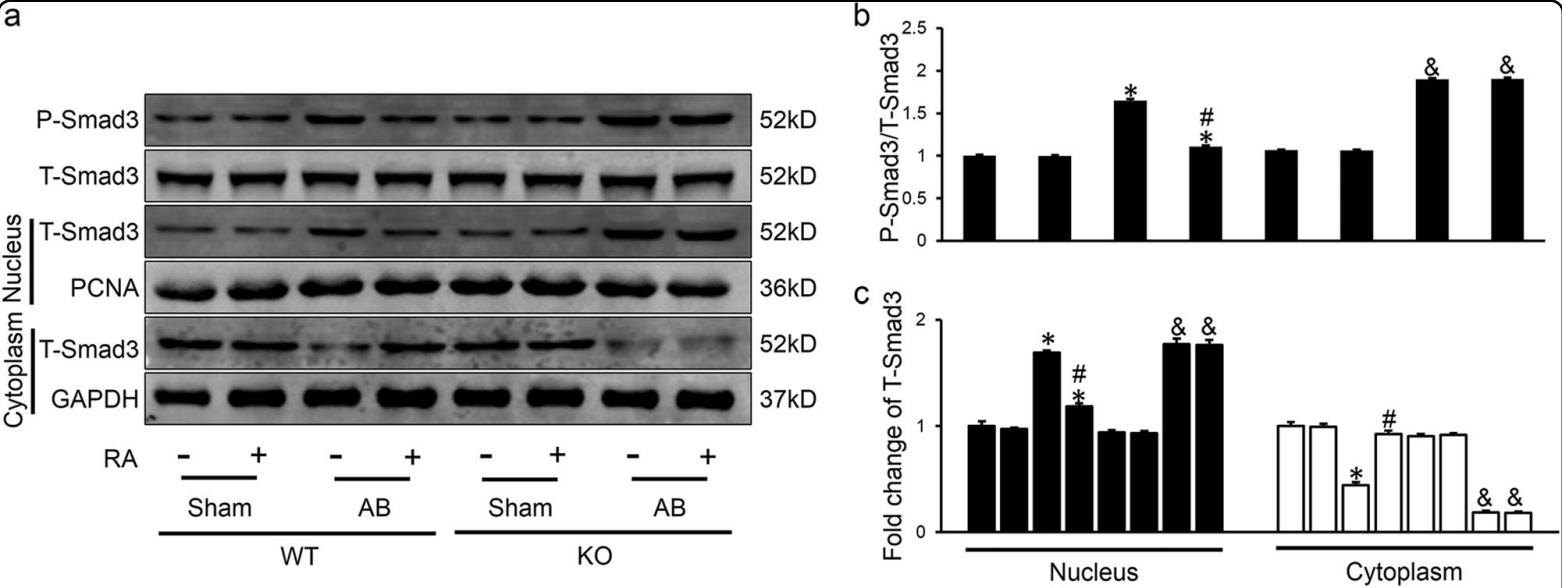
RA suppressed Smad3 phosphorylation and nuclear translocation through AMPKα activation **a**-**c** Representative western blots and statistical results (*n* = 6). Values represent the mean ± SEM. *P < 0.05 vs. the corresponding Sham group within WT mice, ^#^*P* < 0.05 vs. AB+Veh in WT mice, ^&^*P* < 0.05 vs. the corresponding Sham group in KO mice

Intensive investigation is underway to shed light into the mechanisms of RA in ameliorating cardiac fibrosis. It is well recognized that Smad3 is a critical and necessary mediator of cardiac fibrosis in response to pressure overload^8,35^, we then examined the effect of RA on the phosphorylation and nuclear translocation of Smad3, which are important for determining the strength and duration of the signal and biological response^8,36^. Increased phosphorylation of Smad3 was observed after pressure overload, which was prevented after the treatment of RA (Figs. 4a, b). After sub-fractionating left ventricles into cytosolic fraction and nuclear fraction, we found that increased protein level of Smad3 in nuclear fraction and decreased level in cytosolic fraction after AB operation were reversed by RA (Figs. 4a, c). RA alone showed no influence on phosphorylation or nuclear translocation of Smad3 in sham surgery (Fig. 4a-c). And previous study showed that activation of AMPKα could inhibit phosphorylation and nuclear translocation of Smad3^14^, we further investigated that whether AMPKα was involved in the regulation on Smad3 of RA, western blot analysis showed that inhibitory effect of RA on Smad3 was counteracted in AMPKα2 KO mice (Figs. 4a-c).

### RA blocked transdifferentiation of CFs via AMPKα/Smad3 signaling pathway in vitro

**Fig. 5:**
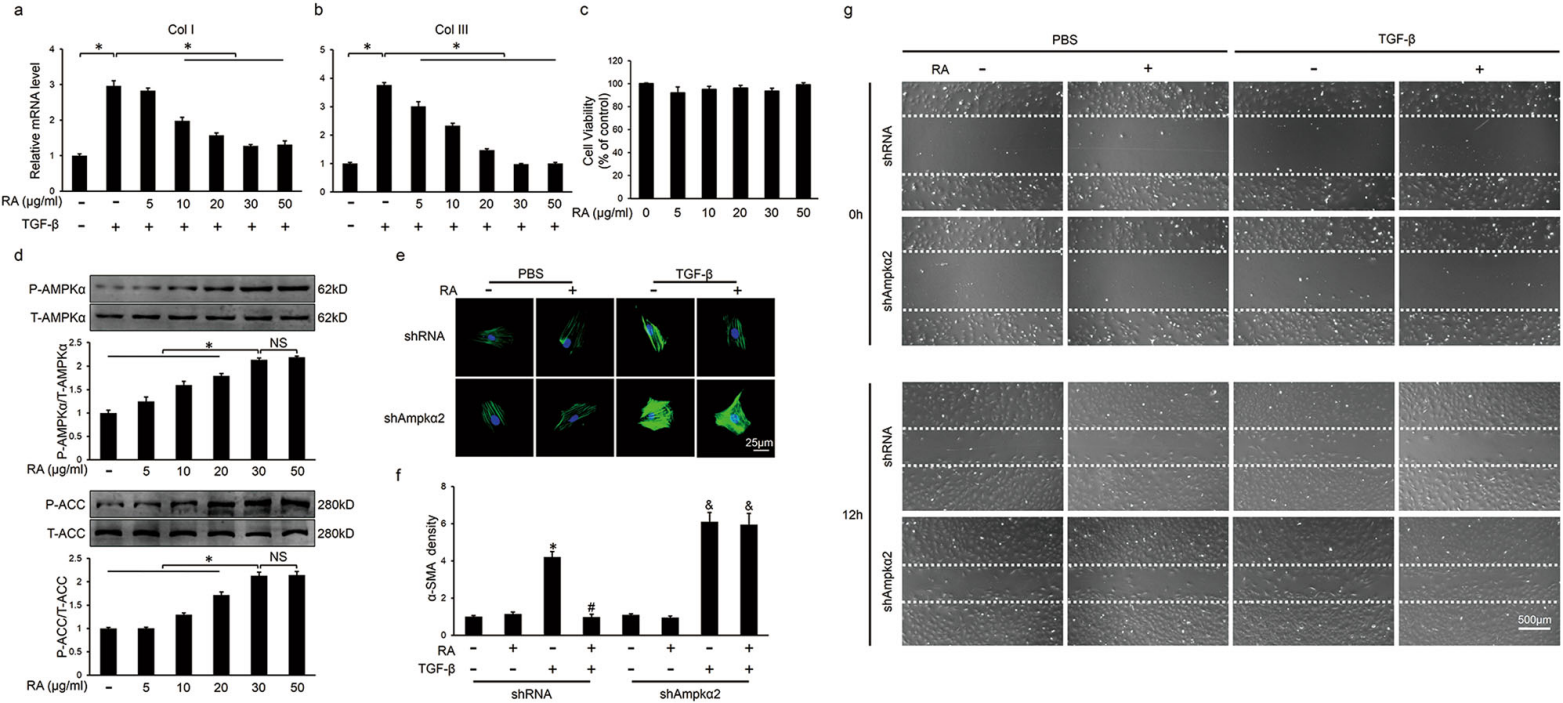
RA blocked transdifferentiation of neonatal rat cardiac fibroblasts (CFs) via AMPKα in vitro **a**, **b** The relative mRNA levels of collagen I (Col I) and collagen III (Col III) in CFs (*n* = 3). **c** Statistical results on cell viability evaluated by cell count kit 8 (*n* = 5). **d** Representative western blots and statistical results (*n* = 6). **e,** **f** Representative immunofluorescence staining of α-SMA in the presence or absence of shAmpkα2 and statistical results (*n* = 6). Green represented α-SMA, nuclei was stained with DAPI (blue). **g** Representative images of wound scratch assay at 0 and 12 h (*n* = 3). Values represent the mean ± SEM of three independent experiments. *P < 0.05 vs. the corresponding PBS-treated group within shRNA-treated CFs, ^#^*P* < 0.05 vs. TGF-β-treated CFs within shRNA group, ^&^*P* < 0.05 vs. the corresponding PBS-treated CFs in shAmpkα2 group. In Figs. 5a, b and 5d, *P < 0.05 vs. the matched group, NS no significance

**Fig. 6:**
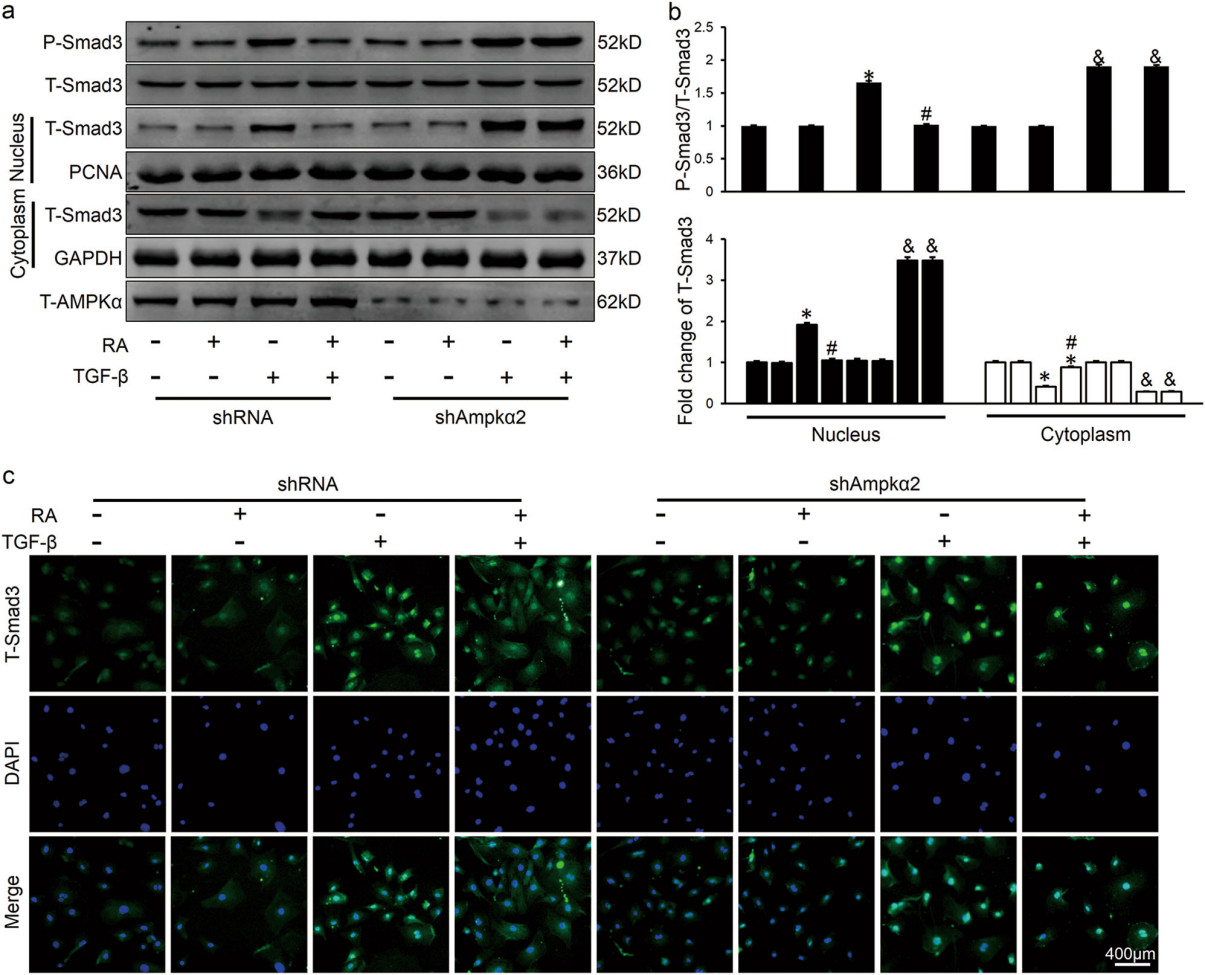
RA suppressed Smad3 phosphorylation and nuclear translocation through activating AMPK in vitro **a,** **b** Representative western blots and statistical results. CFs were pretreated with shAmpkα2 or shRNA for 4 h at a MOI of 50, incubated with RA for 30 min and then stimulated with TGF-β (10 ng/ml) for additional 1 h (*n* = 6). **c** Characteristic immunofluorescence images of T-Smad3 distribution (*n* = 6). CFs were pretreated with RA for 30 min and then stimulated with TGF-β (10 ng/ml) for additional 1 h. Green represented T-Smad3, nuclei was stained with DAPI (blue). Values represent the mean ± SEM of three independent experiments. *P < 0.05 vs. the corresponding PBS-treated CFs within shRNA group, ^#^*P* < 0.05 vs. TGF-β-treated CFs within shRNA group, ^&^*P* < 0.05 vs. TGF-β-treated CFs in shAmpkα2 group

There is common agreement that CFs are the primary cell type most responsible for cardiac fibrosis during pathological cardiac remodeling and transdifferentiation of CFs to myofibroblasts is a hallmark in cardiac fibrosis. To further assess the effect of RA in vitro, we separated neonatal rat CFs and cardiomyocytes. Consistent with the data in vivo, we found unaffected cross sectional area and undiminished mRNA level of hypertrophic markers after RA treatment (20 μg/ml) in terms of cardiomyocytes in response to phenylephrine (PE) stimuli (Figure S3a-d). As shown in Figs. 5a, b, RA inhibited TGF-β-induced mRNA expression of fibrillar collagen in a dose-dependent manner. Mechanistically, continuous activation of AMPKα in a dose-dependent manner by RA was also observed (Fig. 5d). As the anti-fibrotic effect mediated by RA may be ascribed to its cell damage activity^37^, thus we tested the viability of CFs and no cytotoxicity was found in the concentration gradient used in our experiment (Fig. 5c). RA (30 μg/ml) decreased the mRNA level of fibrotic markers and the expression of α-SMA, all of which were negated after the knockdown of AMPKα2 (Figs. 5e, f, Figure S3e-g). In addition, RA treatment significantly inhibited TGF-β-induced CFs migration and the effect was blocked by AMPK inhibition (Fig. 5g). Our results further confirmed that RA reversed the phosphorylation and nuclear translocation of Smad3, which was blocked in the absence of AMPKα2 (Figs. 6a-c). These results showed that RA blocked transdifferentiation of CFs via AMPKα/Smad3 signaling pathway in vitro, and subsequently decreased the accumulation of ECM.

### RAactivated AMPKα via the activation of PPAR-γ

**Fig. 7:**
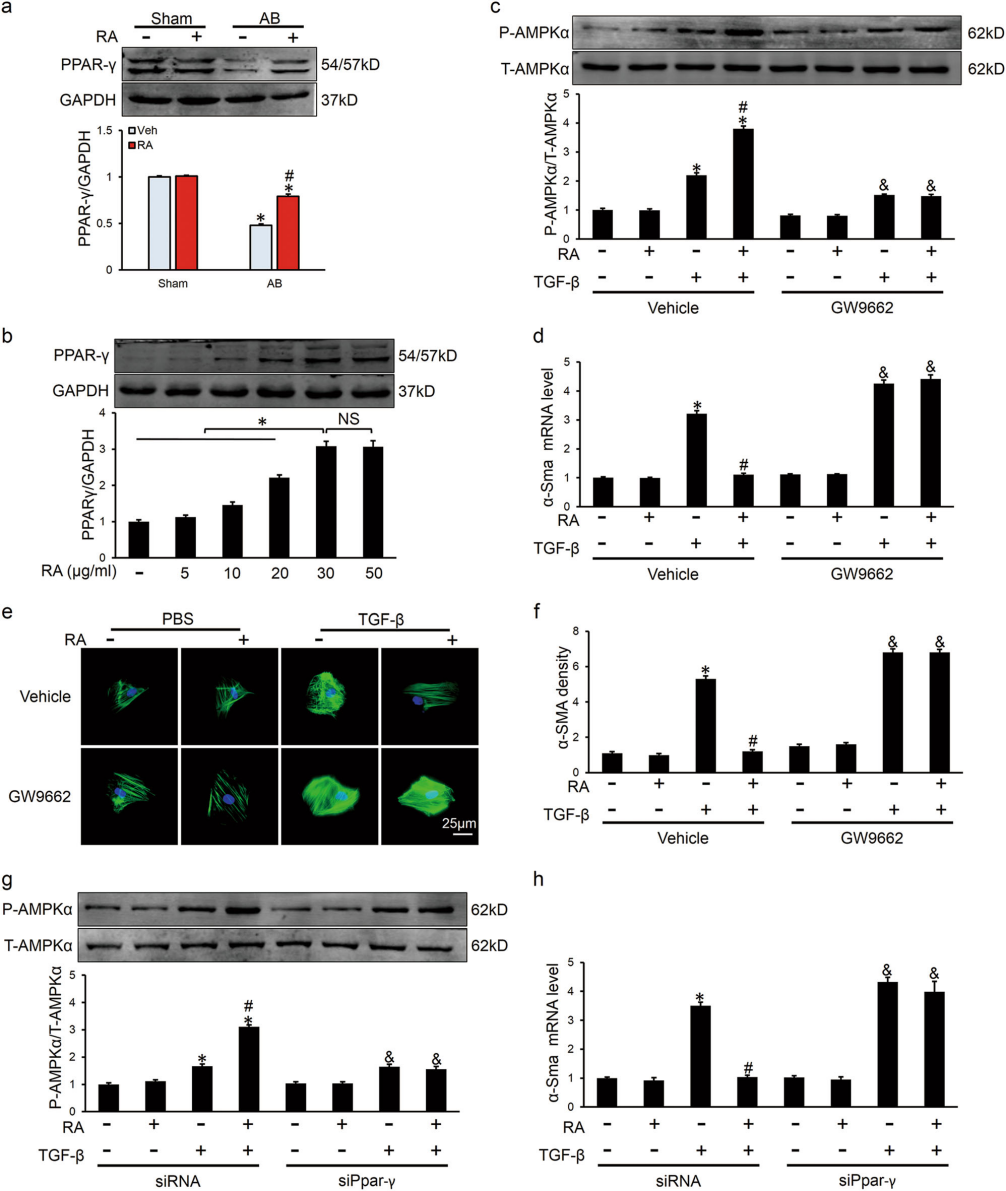
RAactivated AMPKα via the activation of PPAR-γ **a** The expression of PPAR-γ and the quantitative results in vivo (*n* = 6). **b** Representative western blots and statistical results of PPAR-γ in CFs (*n* = 6). **c** Activation of AMPKα was blocked in the presence of GW9662 in CFs (*n* = 6). **d** The relative mRNA level of α-Sma normalized to Gapdh in the presence or absence of GW9662 in CFs (*n* = 6). **e**, **f** Representative immunofluorescence staining of α-SMA and statistical results (*n* = 6). Green represented α-SMA, nuclei was stained with DAPI (blue). **g** Activation of AMPKα was blocked after knockdown of PPAR-γ with siPpar-γ in CFs (*n* = 6). **h** The relative mRNA level of α-Sma normalized to Gapdh with or without siPpar-γ treatment in CFs (*n* = 6).Values represent the mean ± SEM. Representative images are shown in **b**-**h** from three independent experiments. *P < 0.05 vs. the corresponding control CFs within vehicle or siRNA group, ^#^*P* < 0.05 vs. TGF-β-treated CFs within vehicle or shRNA group, ^&^*P* < 0.05 vs. the corresponding control CFs within GW9662 or siPpar-γ group. In Figs. 7a, *P < 0.05 vs. the corresponding Sham group, ^#^*P* < 0.05 vs. AB+Veh. In Figs. 7b, *P < 0.05 vs. the matched group, NS no significance

Finally, we investigated the possible mechanism by which RA-activated AMPKα. Previous study showed that RA could epigenetically de-repress Ppar-γ in hepatic stellate cells for the anti-fibrotic effect^24^, and we observed that mRNA and protein levels of PPAR-γ were decreased after AB, which were restored after RA treatment in vivo (Fig. 7a, Figure S4a). In addition, RA increased mRNA level and protein expression of PPAR-γ in a dose-dependent manner in vitro, which aligns with AMPKα activation (Figs. 5d, 7b, Figure S4b). To evaluate the necessity of PPAR-γ in RA-activated AMPKα, we treated CFs with GW9662, and found that activation of AMPKα was inhibited (Fig. 7c), followed by an aggravating fibrotic response (Figs. 7d-f). Knockdown of PPAR-γ with small interfering RNA against Ppar-γ (siPpar-γ) further confirmed our hypothesis (Figs. 7g, h, Figure S4c). Thus, we concluded that RA might activate AMPKα via the activation of PPAR-γ.

## Discussion

In the present study, we found that RA attenuated cardiac dysfunction and cardiac fibrosis following long-term pressure overload. Supplementation of RA blocked transdifferentiation of CFs and inhibited accumulation of collagen in TGF-β-treated CFs. AMPKα suppressed phosphorylation and nuclear translocation of Smad3, and these protective effects were abolished by AMPKα deficiency in vivo and in vitro. And we also found that PPAR-γ was essential for the activation of AMPKα. Thus, our current findings identified RA as a novel therapeutic agent against cardiac fibrosis.

Increasing pieces of evidence indicated a possible role for RA on cardiac remodeling due to its pleiotropic bioactivities, including powerful anti-oxidative and anti-inflammatory effects^19,31,38^. RA could decrease oxidative stress-induced cell damage via activating Nrf2/antioxidant response element signaling pathway^31^. The inhibitory effect on nuclear factor kappa-light chain-enhancer of activated B cells activation could reduce inflammation response^39^. However, our study demonstrated that RA exerted a protective effect on cardiac fibrosis independent of the aforementioned factors. Obviously, excessive deposition of ECM results in profound structural and functional abnormalities of the heart. Clinical researches disclosed that late gadolinium enhancement defined fibrosis was associated with occurrence of sudden cardiac death, high rate of future cardiovascular event, and heart failure hospitalization^40,41^, suggesting a deleterious role of cardiac fibrosis and an urgent need for pharmacological interventions. The currently available pharmacological treatment for cardiac fibrosis, including agents targeting renin-angiotensin-aldosterone system or sympathetic nerves system, achieved certain effects, but their applications are limited in some clinical conditions due to their side effects. Thus, it is of great significance that our results collectively provided RA as an anti-fibrotic candidate in the prevention and treatment of cardiac fibrosis.

AMPK is a highly conserved eukaryotic serine/threonine protein kinase, mainly known as an energy sensor in regulating energy homeostasis; however, current available studies showed that its actions extended well beyond its energy-regulating function and it appeared to play an essential role in regulating cardiac fibrosis^42,43^. Activation of AMPK led to suppressed cardiac fibrosis and improved cardiac function^12,44^, whereas deletion of AMPK was detrimental for cardiac function because of the aggravated fibrosis^11^. And our previous study also indicated that activation of AMPK prevented pressure overload induced cardiac fibrosis^45^. Herein, we found that RA prevented against cardiac fibrosis via activating AMPK and KO of AMPK abrogated the protective effect, which is consistent with a previous study showing that RA-triggered activation of AMPK was responsible for its inhibition on insulin resistance in skeletal muscle cells^46^. The data in our study indicated a key role of AMPK in RA-mediated cardiac protection.

Smad3 is a key transcription factor that mediates the pathogenesis of cardiac fibrosis and targeting Smad3 could inhibit the initiation and progression of cardiac fibrosis^8^. Once phosphorylated and activated, Smad3 forms a complex with co-Smad and translocates to the nucleus, where it binds to specific *cis*-elements and enhances the expression of fibrillar collagen^9^. Huang et al. showed that KO of Smad3 in mice protected against cardiac fibrosis induced by angiotensin II infusion^10^. In the present study, we showed that RA suppressed phosphorylation and nuclear translocation of Smad3 in an AMPK-dependent manner, which is consistent with a previous study^14^. In contrast, Rangnath et al. demonstrated that in TGF-β-induced human primary mesangial cells and murine embryonic fibroblasts, AMPK blocked Smad3-mediated transcription of fibrosis-related genes by inhibiting its binding to the promoter of the target gene but did not inhibit Smad3 phosphorylation or nuclear translocation^47^. Taken together, we found that the protective effect of RA on cardiac fibrosis was mediated partially, if not fully, through the activation of AMPK and the inhibition of Smad3.

PPAR-γ, a member of the nuclear receptor superfamily, is regulated by direct binding of steroid and thyroid hormones, vitamins, and lipid metabolites, etc. Recent evidence identified RA as an activator of PPAR-γ involving the MeCP2-EZH2 relay via Wnt signaling^24^. We found that PPAR-γ was essential for the activation of AMPK by RA, in line with previous data showing that endothelial cell survival and function preservation was relied on PPAR-γ-dependent activation of AMPK^48^. PPAR-γ is primarily expressed in adipose tissue, where it is the key orchestrator of the transcriptional cascade mediating adipocyte differentiation, lipid metabolism, and adiponectin secretion^49,50^. Increased amount of adiponectin derived from mature adipocytes is considered as a direct activator of AMPK. A recent study also defined FGF21 as a crucial mediator linking PPAR-γ to AMPK^51^. Additionally, thiazolidinediones, agonists of PPAR-γ, have been proven to be effective in activating AMPK^25,26^. Collectively, these findings further verify our result that PPAR-γ is essential for the activation of AMPK by RA.

In summary, the present study indicated that supplementation of RA attenuated cardiac dysfunction, as well as inhibited cardiac fibrosis following long-term pressure overload in vivo, and blocked transdifferentiation of CFs in vitro. The beneficial effects of RA on fibrotic response may be ascribed to its activation of AMPK and inhibition of Smad3. Our research collectively provided RA as a candidate in the prevention and treatment of cardiac fibrosis.

## Materials and methods

### Antibodies and reagents

RA (≥98% purity, as confirmed by high-performance liquid chromatography) was obtained from Shanghai Winherb Medical Co. (Shanghai, China, no. 160603) and solubilized in 0.1% dimethylsulfoxide (DMSO). TGF-β (T7039) and PE (P6126) were purchased from Sigma-Aldrich (St. Louis, MO, USA). Replication-defective vectors carrying small hairpin RNA against AMPKα2 (shAmpkα2) were obtained from Vigene Bioscience (Rockville, MD, USA), and scramble (short hairpin RNA (shRNA)) was from Sigma-Genosys (Spring, TX, USA). The efficacy of shAmpkα2 has been verified by our previous studies^45,52^. Small interfering RNAs (siRNAs) targeting Ppar-γ were generated by RiboBio (RiboBio Co., Ltd, Guangzhou, China). Three siRNAs were generated and the one resulting in the most downregulated expression of PPAR-γ was used for further studies. Primary antibodies against the following proteins were purchased from Cell Signaling Technology (Danvers, MA, USA): SOD2 (13141s), COX2 (12282), protein kinase B (T-AKT, 4691), P-AKT (4060), p38 mitogen-activated protein kinase (T-P38, 9212P), P-P38 (4511P), extracellular signal-regulated kinase (T-ERK, 4695), P-ERK (4370P), T-AMPKα (2603P), P-AMPKα (2535), acetyl-CoA carboxylase (T-ACC, 3676P), P-ACC (3661P), T-Smad3 (9513S), P-Smad3 (8769) and glyceraldehyde 3-phosphate dehydrogenase (GAPDH, 2118). Antibodies for Nrf2 (ab31163), iNOS (ab3523), α-SMA (ab5694), α-actinin (ab68167), vimentin (ab92547) and TGF-β1 (ab64715) were obtained from Abcam (Cambridge, UK). Anti-Col I (14695-1-AP), anti-Col III (13548-1-AP), and anti-CTGF (23936-1-AP) were purchased from Proteintech (Manchester, UK), whereas anti-proliferating cell nuclear antigen (PCNA, sc-7907, 1:200 dilution) and anti-PPAR-γ (sc-7196, 1:200 dilution) were purchased from Santa Cruz Biotechnology (Dallas, TX, USA). The GTVision^TM^+Detection System/Mo&Rb reagent for immunohistochemistry was purchased from Gene Technology (Shanghai, China) and Alexa Fluor 488-goat anti-rabbit secondary antibody for immunofluorescence staining was obtained from LI-COR Biosciences (Lincoln, USA). All antibodies were used at a dilution of 1:1000 if not otherwise indicated. The BCA protein assay kit was from Pierce (Rockford, IL, USA). All other chemicals were of analytical grade.

### Animals and treatments

All animals were given humane care in compliance with the Animal Care and Use Committee of Renmin Hospital of Wuhan University, which is also in agreement with the instruction of Guidelines for the Care and Use of Laboratory Animals published by the United States National Institutes of Health (NIH Publication, revised 2011). A blinded manner ran through the entire experimental process all along, including the surgeries and subsequent analyses. The groups of mice were revealed upon destination of the experiment and the data were obtained and analyzed by two independent individuals, respectively, without a prior knowledge of the hypothesis and interventions in this study.

Male C57/B6 mice (age: 8–10 weeks old; BW: 25.5 ± 2 g) were purchased from the Institute of Laboratory Animal Science, Chinese Academy of Medical Sciences (Beijing, China). The animals were allowed access to food and drinking water ad libitum and were fed on a 12-h light/dark cycle with a controlled temperature (20–25 °C) and humidity (50 ± 5%) environment for a period of 1 week before the study commenced. The AB model was employed to generate pressure overload induced cardiac remodeling as described previously^53^. Briefly, mice were anesthetized with 3% pentobarbital sodium (50 mg/kg, Sigma) by intraperitoneal injection and randomly assigned to AB surgery or sham-operated control group. Under general anesthesia, the thoracic aorta was surgically dissected at the second intercostal space after left hemithoracotomy. Then a 27-G blunt needle was placed along the side of the isolated aorta segment, with a 7-0 silk suture tightly tied around the aorta and the overlying needle. The needle was then removed, thus producing severe aortic constriction. Meanwhile, animals assigned to the sham-operated control group underwent the same procedures without actual ligation of the aorta. Temgesic (qd, 0.1 mg/kg) was used subcutaneously for postoperative pain relief. One week after operation, after confirmation for the adequate ligation via Doppler analysis, mice were intragastrically administered (09:00 am) with RA (100 mg/kg) once daily referring to previous studies^54,55^ or isovolumic vehicle for 7 weeks. Thus, all the mice were divided into four groups randomly: sham+vehicle, sham+RA, AB+vehicle, and AB+RA (*n* = 15 per group). After the experimental period, mice were sacrificed with heart and tibia collected for calculating HW/BW and HW/TL ratios and elaborating the underlying mechanisms. Mice were orally administered RA (100 mg/kg/d) for 3 weeks beginning at one week post-surgery to explore the effect of RA on inflammatory response in vivo. To ascertain whether the beneficial effect of RA on cardiac fibrosis was mediated by AMPKα, AMPKα2 KO mice were used. The source of AMPKα2 global KO mice has been described previously^45^.

### Echocardiography and hemodynamics

Transthoracic echocardiography was performed by a MyLab 30CV ultrasound (Esaote SpA, Genoa, Italy) with a 10 MHz phased array transducer as previously described^56,57^. Light anesthetized by 1.5% isoflurane, mice were lightly secured in a warming pad with a shallow left lateral position after the precordium shaved. After imaged with two-dimensional (2D) mode in the parasternal long-axis and/or parasternal short-axis at the level close to papillary muscles, a 2D guided M-mode trace crossing the anterior and posterior wall of the left ventricle was recorded and the morphological parameters of the heart were collected and calculated. Attention was given not to bring excessive pressure to the chest, which could cause bradycardia and deformation of the heart.

Invasive hemodynamic monitoring was performed by cardiac catheterization according to our previous articles^56,57^. In brief, mice was placed on a warmed surgical platform with supine position and the right carotid artery was isolated, exposed. Then a 1.4-French Millar catheter transducer (SPR-839; Millar Instruments, Houston, TX) was inserted into the left ventricle through the isolated carotid artery. And data were analyzed using the PVAN data analysis software.

### Histological analysis and immunohistochemistry

Fixed hearts were dehydrated and embedded in paraffin. In all, 5-μm slices sectioned from middle segment of the heart were performed to HE for the examination of overall morphology and calculation of cardiomyocyte area, and PSR staining was determined for evaluating collagen deposition. Data were analyzed by a digital analysis software (Image-Pro Plus 6.0, Media Cybernetics, Bethesda, MD, USA) with 50 cells per slide analyzed for the detection of the cardiomyocyte area and >60 fields per group assessed for the evaluation of fibrosis.

Immunohistochemical staining was determined for further assessing the extent of cardiac fibrosis by GTVision^TM^+Detection System/Mo&Rb (GK600710) according to the manufacturer′s protocol. Endogenous peroxidase and the nonspecific binding of the antibody were blocked with 3% hydrogen peroxide for 20 min and 10% goat serum for 45 min, respectively, at room temperature. And slices were incubated with the indicated primary antibodies (1:100) overnight at 4 °C and with GTVision^TM^+Detection System/Mo&Rb reagent for 60 min at 37 °C. Then, the slices were examined by the light microscopy after being visualized with diaminobenzidin for 2 min at room temperature.

### Western blot and quantitative real-time PCR

After being extracted from the frozen pulverized left ventricles or cultured cells and quantified, total proteins were prepared for western blot analysis and normalized to the matched total proteins or GAPDH according to our previous study^52^. Briefly, separated proteins were incubated with the indicated primary antibodies overnight at 4 °C and with secondary antibodies for 60 min at room temperature. Nuclear and cytosolic protein fractions were separated using a commercial kit (Thermo Fisher Scientific) according to the manufacturer′s protocol. Proteins from cytosolic lysates were normalized to GAPDH, whereas proteins from nuclear lysates were normalized to PCNA.

Isolated total mRNA from hearts and cultured cells was reversely transcribed to complementary DNA (cDNA) with Transcriptor First Strand cDNA Synthesis Kit (Roche (Basel, Switzerland), 04896866001). Transcriptional level of target genes were normalized to Gapdh, and the primers for quantitative real-time PCR are shown in Supplementary Table 3.

### Cell culture, treatments, and infection

Neonatal rat CFs and cardiomyocytes were prepared by the methods according to previous literatures^58,59^. Bromodeoxyuridine (0.1 mM) was employed to inhibit proliferation of CFs in neonatal rat cardiomyocytes. Cardiomyocytes were seeded into 6- and 24-well plates and cultured in Dulbecco’s modified Eagle’s medium (DMEM)/F12 (GIBCO, C11995) supplemented with 10% fetal bovine serum (FBS, GIBCO, 10099) for 48 h. After grown to 70–80% confluency and serum deprived for 16 h to synchronize, cardiomyocytes were assigned randomly to incubate with RA (20 μg/ml)^21^ or equal volume of vehicle for 24 h in the presence or absence of PE (50 μM). Eventually, cells were collected for western blot, quantitative real-time PCR, and immunofluorescence staining without awareness of the sample group allocation during the experiment.

The purity of cultured CFs was measured by a negative result of anti-α-actinin and positive result of anti-vimentin via immunofluorescence staining. CFs in passages 2 and 3 were used for all studies. After cultured in DMEM/F12 with 15% FBS for 48 h and synchronization, CFs were randomly treated with the indicated concentration of RA with or without TGF-β (10 ng/ml) stimulation for 24 h. To investigate the effect of RA on the phosphorylation and nuclear translocation of Smad3, CFs seeded in six-well plates or coverslips were pretreated with RA (30 μg/ml) for 30 min before addition of TGF-β (10 ng/ml) to the medium and incubation for an additional 1 h^14^. RA was dissolved in 0.1% DMSO as a stock solution (20 mg/ml) and diluted to the desired final concentrations. For evaluating the effect of RA on the proliferation capacity of CFs, cell counting kit (CCK-8; Dojindo Molecular Technologies, Rockville, MD, USA) was used referring to the manufacturer′s protocol.

We knocked down the expression of AMPKα2 via shAmpkα2 carried by adenovirus as our previous study^52^. In brief, CFs were infected with shAmpkα2 or shRNA for 4 h at a multiplicity of infection (MOI) of 50, then rinsed and synchronized with serum-free medium for 16 h before further studies.

For PPAR-γ inhibition, CFs were treated with GW9662 (10 μM), a specific antagonist of PPAR-γ, for 24 h prior to interventions^60^. Moreover, genetic ablation targeting Ppar-γ with small interfering RNA (siPpar-γ) was performed to knockdown the expression of PPAR–γ referring to a previous study^61^.

### Wound scratch assay of primary CFs

Wound scratch assay was performed according to a previous study^62^. Briefly, CFs were passaged in 24-well plates and were cultured to form a confluent monolayer, and then, a scratch in the cell monolayer was generated by using a 200 μl sterilized micropipette tip. After being gently rinsed with phosphate-buffered saline (PBS) for three times, CFs were immediately treated with RA (30 μg/ml) or vehicle in the presence or absence of TGF-β (10 ng/ml) and were cultured with serum-free medium to exclude the influence of cell proliferation. The cells were photographed before and at 12 h after treatment using inverted microscope (IX51, Olympus, Tokyo, Japan).

### Immunofluorescence staining

Immunofluorescence staining was performed as previously described^52,60^. Briefly, cardiac slices or cell coverslips were fixed with 4% formaldehyde, permeabilized in 0.2% Triton X-100, and stained with α-actinin (1:100), α-SMA (1:100), or T-Smad3 (1:75) after being blocked with 10% goat serum for 60 min at 37 °C, and followed with a Alexa Fluor 488-goat anti-rabbit secondary antibody (1:200), after which slices and coverslips were visualized, with 4,6-diamidino-2-phenylindole (DAPI) used for nuclei observation, via a special OLYMPUS DX51 fluorescence microscope. Images were analyzed via Image-Pro Plus 6.0 in a blind manner.

### Statistical analysis

Results in our study are presented as the mean ± standard error of the mean (SEM). Differences between two groups were analyzed by unpaired, two tailed-Student′s *t*-tests. To evaluate the effects of RA on AB, two-way analysis of variance (ANOVA) followed by Bonferroni′s post hoc test was used. One-way ANOVA was carried out to compare differences among three or more groups, followed by post hoc Tukey test. *P* < 0.05 was considered as an indicator of statistical significance.

## Electronic supplementary material

Supplementary Information

Supplementary Figure S1

Supplementary Figure S2

Supplementary Figure S3

Supplementary Figure S4
