# Supplementary material for: Rosmarinic acid attenuates cardiac fibrosis following long-term pressure overload via AMPKα/Smad3 signaling
Source: Cell Death Dis. 2018 Jan 24;9(2):102. doi: 10.1038/s41419-017-0123-3 (PMC5833382; doi:10.1038/s41419-017-0123-3)
Supplement: Supplementary file 1 — Supplementary Information [file 41419_2017_123_MOESM1_ESM.docx]

**Rosmarinic Acid Attenuates Cardiac Fibrosis Following Long-Term Pressure Overload via AMPKα/Smad3 Signaling**

Xin Zhang^*^, Zhen-Guo Ma^*^, Yu-Pei Yuan, Si-Chi Xu, Wen-Ying Wei, Peng Song, Chun-Yan Kong, Wei Deng & Qi-Zhu Tang

Department of Cardiology, Renmin Hospital of Wuhan University, Wuhan 430060, RP China

Cardiovascular Research Institute of Wuhan University, Wuhan 430060, RP China

Hubei Key Laboratory of Cardiology, Wuhan 430060, RP China

^*^ These authors contributed equally to this work.

Corresponding author:

**Qi-Zhu Tang**,

Department of Cardiology,

Renmin Hospital of Wuhan University,

Cardiovascular Research Institute,

Hubei Key Laboratory of Cardiology,

Wuhan University at Jiefang Road 238, Wuhan 430060, RP China

Tel.: +86 27 88073385; Fax: +86 27 88042292. E-mail: [qztang@whu.edu.cn](mailto:qztang@whu.edu.cn)

**Wei Deng,**

Department of Cardiology,

Renmin Hospital of Wuhan University,

Cardiovascular Research Institute,

Hubei Key Laboratory of Cardiology,

Wuhan University at Jiefang Road 238, Wuhan 430060, RP China

Tel.: +86 27 88073385; Fax: +86 27 88042292. E-mail: vivideng1982@whu.edu.cn

Running title: Rosmarinic Acid Attenuates Cardiac Fibrosis

The authors declare no conflict of interest.

**Supplementary Table 1: All echo data for different groups in the first animal experiment.**

|  | Sham+Veh  (n=15) | Sham+RA  (n=15) | AB+Veh  (n=15) | AB+RA  (n=15) |
| --- | --- | --- | --- | --- |
| HR (bpm) | 480.60±13.00 | 477.13±15.72 | 478.13±17.48 | 463.27±9.82 |
| FS (%) | 44.20±0.72 | 46.67±0.93 | 26.73±2.15^*^ | 34.67±0.99^*#^ |
| EF (%) | 75.87±0.75 | 75.73±0.90 | 53.73±1.18^*^ | 66.40±1.25^*#^ |
| LVIDd(mm) | 3.93±0.04 | 4.05±0.09 | 4.59±0.13^*^ | 4.13±0.06^#^ |
| LVIDs(mm) | 2.19±0.03 | 2.17±0.04 | 3.36±0.12^*^ | 2.70±0.05^*#^ |
| IVSd(mm) | 0.79±0.01 | 0.78±0.02 | 0.85±0.02^*^ | 0.85±0.03^*^ |
| IVSs(mm) | 1.09±0.02 | 1.08±0.02 | 1.25±0.05^*^ | 1.22±0.03^*^ |
| LVPWd(mm) | 0.80±0.02 | 0.80±0.03 | 0.87±0.02^*^ | 0.85±0.03 |
| LVPWs(mm) | 1.21±0.07 | 1.21±0.06 | 1.25±0.05 | 1.24±0.04 |

HR, heart rate; FS, fractional shortening; EF, ejection fraction; LVIDd, left ventricular end diastolic dimension; LVIDs, left ventricular end systolic dimension; IVSd, interventricular septal thickness at diastole; IVSs, interventricular septal thickness at systole; LVPWd, left ventricular posterior wall thickness at end-diastole; LVPWs, left ventricular posterior wall thickness at end-systole. Values represent the mean±SEM. **P*<0.05 versus the corresponding Sham group, #*P*<0.05 versus AB+Veh.

**Supplementary Table 2: All echo data for different groups in the second animal experiment.**

|  | WT | | | |  | KO | | | |
| --- | --- | --- | --- | --- | --- | --- | --- | --- | --- |
|  | Sham+Veh  (n=9) | Sham+RA  (n=9) | AB+Veh  (n=10) | AB+RA  (n=10) |  | Sham+Veh  (n=8) | Sham+RA  (n=8) | AB+Veh  (n=8) | AB+RA  (n=8) |
| HR (bpm) | 477.33±13.78 | 478.56±17.27 | 470.00±15.08 | 476.20±15.46 |  | 476.75±17.88 | 486.25±19.88 | 485.50±20.63 | 483.25±24.63 |
| FS (%) | 44.56±1.24 | 45.22±1.36 | 26.30±1.28^*^ | 40.50±1.54^*#^ |  | 45.13±1.87 | 44.88±1.88 | 22.88±1.09^&^ | 22.25±0.84^&^ |
| EF (%) | 73.78±1.93 | 74.78±1.20 | 53.90±2.84^*^ | 66.20±2.89^*#^ |  | 71.88±1.80 | 72.00±2.07 | 45.88±2.14^&^ | 44.75±1.53^&^ |
| LVIDd(mm) | 3.89±0.06 | 3.89±0.06 | 4.54±0.05^*^ | 4.13±0.10^*#^ |  | 3.89±0.12 | 3.95±0.07 | 4.58±0.12^&^ | 4.51±0.05^&^ |
| LVIDs(mm) | 2.16±0.05 | 2.12±0.05 | 3.35±0.09^*^ | 2.44±0.03^*#^ |  | 2.13±0.05 | 2.18±0.07 | 3.53±0.11^&^ | 3.50±0.05^&^ |
| IVSd(mm) | 0.79±0.02 | 0.79±0.03 | 0.88±0.01^*^ | 0.87±0.02^*^ |  | 0.79±0.02 | 0.79±0.02 | 0.93±0.02^&^ | 0.92±0.01^&^ |
| IVSs(mm) | 1.12±0.03 | 1.13±0.02 | 1.28±0.02^*^ | 1.28±0.02^*^ |  | 1.15±0.02 | 1.11±0.02 | 1.36±0.03^&^ | 1.35±0.02^&^ |
| LVPWd(mm) | 0.79±0.03 | 0.81±0.02 | 0.87±0.03^*^ | 0.87±0.03^*^ |  | 0.80±0.03 | 0.81±0.02 | 0.94±0.01^&^ | 0.94±0.01^&^ |
| LVPWs(mm) | 1.12±0.06 | 1.12±0.09 | 1.25±0.06 | 1.22±0.07 |  | 1.11±0.04 | 1.14±0.07 | 1.50±0.03^&^ | 1.49±0.03^&^ |

HR, heart rate; FS, fractional shortening; EF, ejection fraction; LVIDd, left ventricular end diastolic dimension; LVIDs, left ventricular end systolic dimension; IVSd, interventricular septal thickness at diastole; IVSs, interventricular septal thickness at systole; LVPWd, left ventricular posterior wall thickness at end-diastole; LVPWs, left ventricular posterior wall thickness at end-systole. Values represent the mean±SEM. **P*<0.05 versus the corresponding Sham group within WT group, #*P*<0.05 versus AB+Veh within WT group, &*P*<0.05 versus the corresponding Sham group within KO group.

**Supplementary Table3: Primer Sequences used in our study**

| **Gene** | **Species** |  | **Sequence** |
| --- | --- | --- | --- |
| Gapdh | Mouse | Forward | ACTCCACTCACGGCAAATTC |
|  |  | Reverse | TCTCCATGGTGGTGAAGACA |
| Anp | Mouse | Forward | ACCTGCTAGACCACCTGGAG |
|  |  | Reverse | CCTTGGCTGTTATCTTCGGTACCGG |
| Bnp | Mouse | Forward | GCTGCTTTGGGCACAAGATAG |
|  |  | Reverse | GGTCTTCCTACAACAACTTCAG |
| β-Mhc | Mouse | Forward | CCGAGTCCCAGGTCAACAA |
|  |  | Reverse | CTTCACGGGCACCCTTGGA |
| Col I | Mouse | Forward | CCCAACCCAGAGATCCCATT |
|  |  | Reverse | GAAGCACAGGAGCAGGTGTAGA |
| Col III | Mouse | Forward | CCCAACCCAGAGATCCCATT |
|  |  | Reverse | GAAGCACAGGAGCAGGTGTAGA |
| Ctgf | Mouse | Forward | TGTGTGATGAGCCCAAGGAC |
|  |  | Reverse | AGTTGGCTCGCATCATAGTTG |
| Fn | Mouse | Forward | CCGGTGGCTGTCAGTCA GA |
|  |  | Reverse | CCGTTCCCACTGCTGATTTATC |
| Tgf-β1 | Mouse | Forward | ATCCTGTCCAAACTAAGGCTCG |
|  |  | Reverse | ACCTCTTTAGCATAGTAGTCCGC |
| α-Sma | Mouse | Forward | TGGCTCTGTGCAAGAAGCGGAT |
|  |  | Reverse | AATCTGGGCGTTGTCACTGCGT |
| Il-1β | Mouse | Forward | CCGTGGACCTTCCAGGATGA |
|  |  | Reverse | GGGAACGTCACACACCAGCA |
| Il-6 | Mouse | Forward | AGTTGCCTTCTTGGGACTGA |
|  |  | Reverse | TCCACGATTTCCCAGAGAAC |
| Mcp-1 | Mouse | Forward | TGGCTCAGCCAGATGCAGT |
|  |  | Reverse | CCAGCCTACTCATTGGGATCA |
| Tnf-α | Mouse | Forward | CATCTTCTCAAAATTCGAGTGACAA |
|  |  | Reverse | TGGGAGTAGACAAGGTACAACCC |
| Ppar-γ | Mouse | Forward | ATTCTGGCCCACCAACTTCGG |
|  |  | Reverse | TGGAAGCCTGATGCTTTATCCCCA |

**Supplementary figure and figure legends:**


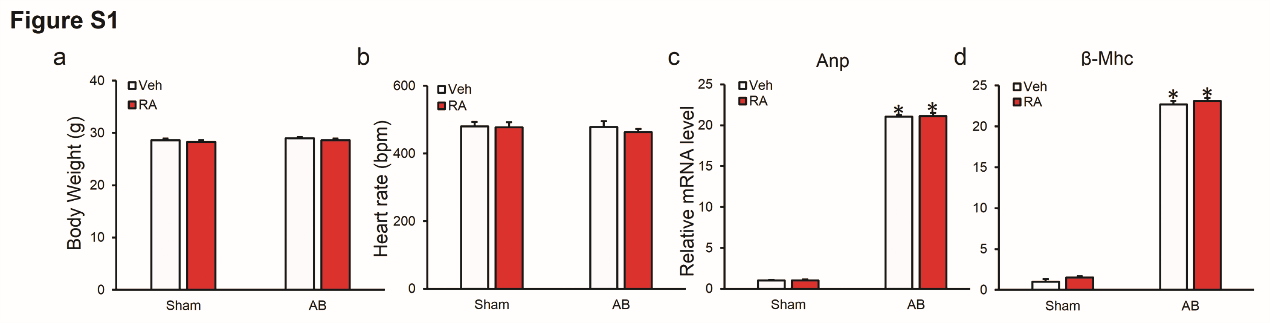


Figure S1. RA attenuated cardiac dysfunction induced by pressure overload with no influence on body weight, heart rate and hypertrophic response. (a) Body weight of mice in response to 8-week operation (n=13-14). (b) Heart rate (n=15). (c-d) The relative mRNA levels of hypertrophic markers normalized to Gapdh (n=6). Values represent the mean±SEM. **P*<0.05 versus the corresponding Sham group.


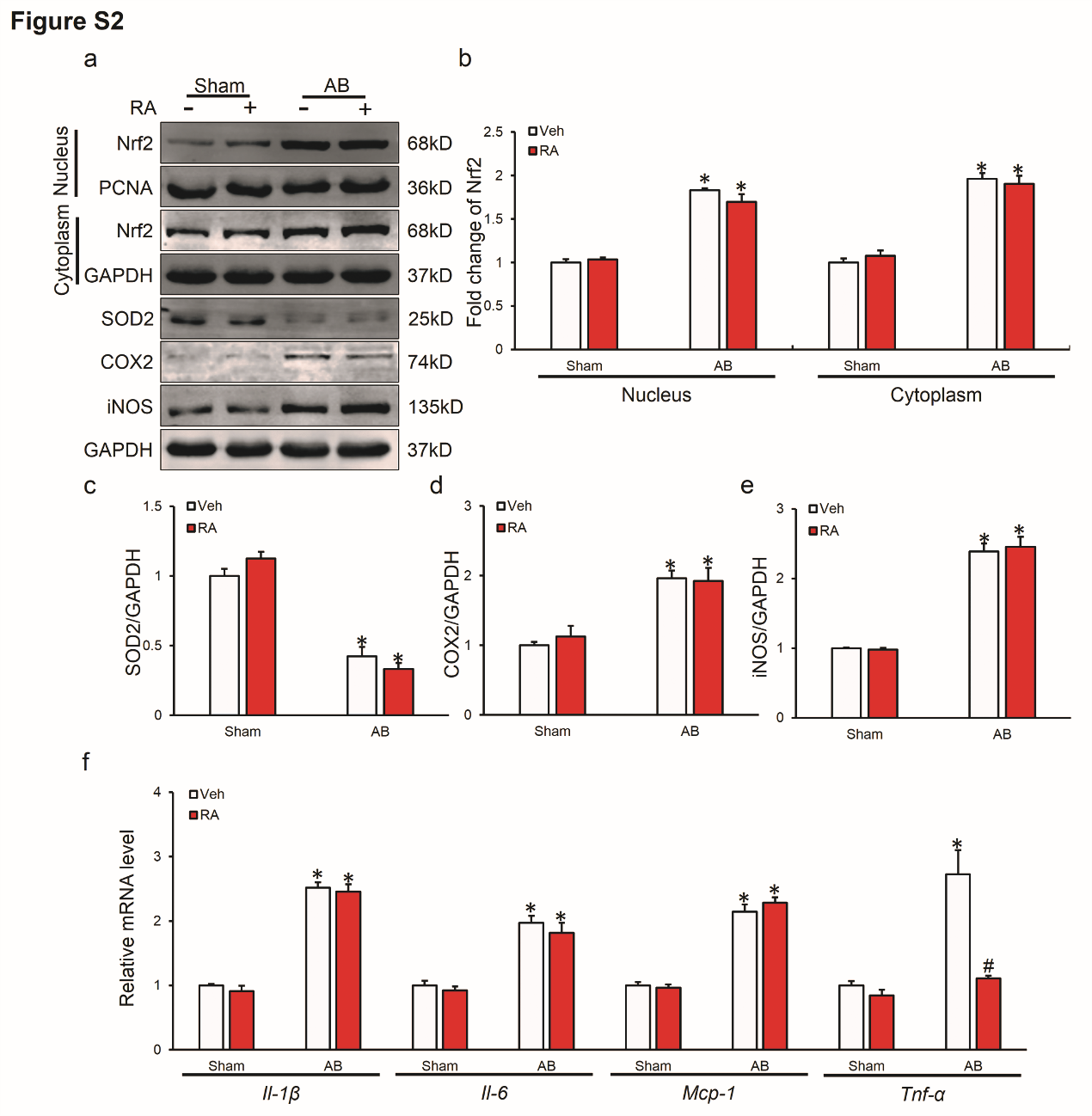


Figure S2. The protective effect of RA on cardiac fibrosis was beyond the inhibition of oxidative stress and inflammation. (a-e) Representative western blots and quantitative results in mice subjected to 8-week operation. The expressions of COX2 and iNOS were detected at 4 weeks after AB surgery (n=6). (f) The relative mRNA levels of markers associated with inflammation in mice subjected to 4-week operation (n=6). Values represent the mean±SEM. **P*<0.05 versus the corresponding Sham group. #*P*<0.05 versus AB+Veh.


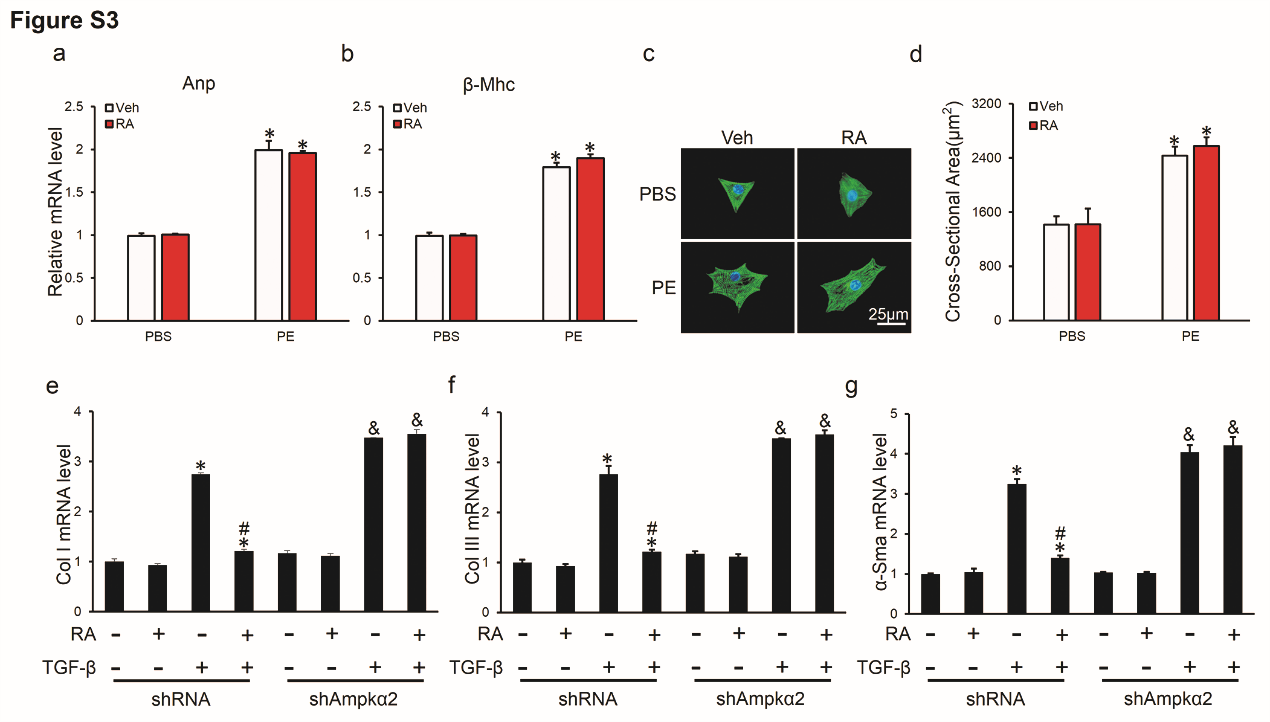


Figure S3. RA prevented against fibrotic responses with no alteration in hypertrophic responses in vitro. (a-b) The relative mRNA levels of hypertrophic markers normalized to Gapdh in cardiomyocytes (n=6). (c-d) Representative images of cardiomyocytes stimulated with phenylephrine (PE, 50μM) and statistical results of cross-sectional area (n=6). Green represented α-actinin, nuclei was stained with DAPI (blue). (e-g) The relative mRNA levels of fibrotic markers normalized to Gapdh in neonatal rat cardiac fibroblasts (CFs) (n=6). Values represent the mean±SEM. All experiments were repeated three times. **P*<0.05 versus the corresponding control group, #*P*<0.05 versus TGF-β stimulated CFs within shRNA group; &*P*<0.05 versus the corresponding control group within shAmpkα2 group.


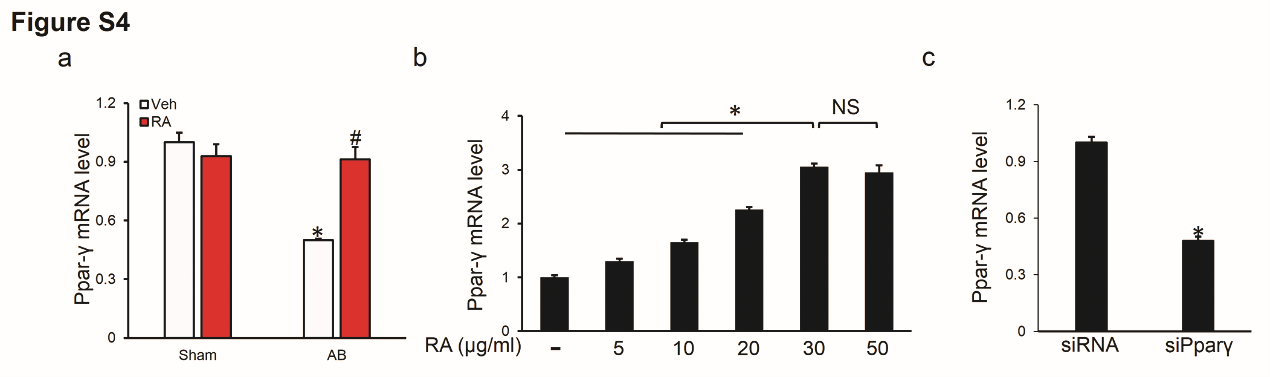


Figure S4. RA activated PPAR-γ in a dose-dependent manner. (a) The relative mRNA level of PPAR-γ normalized to Gapdh in hypertrophic hearts (n=6). (b) The relative mRNA level of PPAR-γ normalized to Gapdh in CFs from three independent experiments (n=6). (c) The efficacy of siPpar-γ was confirmed by PCR (n=6). Values represent the mean±SEM. **P*<0.05 versus the matched control. NS: no significance. In figure S4a, **P*<0.05 versus the corresponding Sham group, #*P*<0.05 versus AB+Veh.
